# Evaluating the Diagnostic Proficiency Among a Sample of Final Stage Dental Students in Some Orthodontic Cases: A Comprehensive Analysis of Clinical Competence

**DOI:** 10.3390/dj13070300

**Published:** 2025-07-02

**Authors:** Noor Nourie Abbass, Zainab Mousa Kadhom, Wurood Khairallah Al-Lehaibi, Mohammed Nahidh

**Affiliations:** 1 College of Dentistry, Al-Iraqia University, Baghdad 10001, Iraq; noor_abbass@aliraqia.edu.iq; 2 Department of Orthodontics, College of Dentistry, University of Baghdad, Baghdad 10001, Iraq; zainabmosa@codental.uobaghdad.edu.iq; 3 Department of Prevention, Orthodontic, Pedodontic Dentistry, Dentistry Department, Dijlah University College, Baghdad 10001, Iraq; wurood.kh@duc.edu.iq

**Keywords:** diagnostic proficiency, dental students, orthodontic cases, clinical competence

## Abstract

**Background/Objectives**: This study evaluates the diagnostic and referral skills of final-year dental students at Al-Iraqia University using a questionnaire based on malocclusion cases ranging from mild to severe. **Methods**: The questionnaire, featuring photos and radiographs of five selected treated cases from two textbooks, was answered by 165 students who were asked to assess each case and determine whether orthodontic or surgical treatment was necessary, as well as to identify factors contributing to an unesthetic profile, such as irregular teeth. Frequency distribution and the Chi-square test were used for statistical analysis. **Results**: The results indicated good overall clinical competence. The unesthetic profile and irregular teeth were the main reasons for referring both Class II and III cases for surgery, with mandibular retrusion being the most common factor in aesthetic concerns. Maxillary protrusion was less frequently selected as a key factor in Class II malocclusion cases. **Conclusions**: The findings suggest that students demonstrated a high level of diagnostic accuracy in identifying treatment needs for various malocclusion cases.

## 1. Introduction

An incorrect relation of the upper and lower teeth is defined as malocclusion [1]. This malocclusion could be shown during function or rest statuses of the dental jaws and during closure, so correct diagnosis and treatment are mandatory for daily functioning, well-being, and overall quality of life to ensure good oral health [2].

A treatment plan should meet the patient’s expectations to improve facial esthetics and correct the malocclusion, along with any dysfunction. Treatment options such as camouflage, functional treatment, or orthognathic surgery are used to treat patients with skeletal discrepancies, depending on the patient’s age, facial esthetics, demand, and the severity of the malocclusion [3].

Continuous analysis of patient treatment results to improve the therapy used and assessment of the credibility of scientific literature allowing decision making are encouraged by the American Dental Association’s information, which indicates the students’ and dentists’ education towards a comprehensive program to the patient, critical thinking, evidence-based treatment, and seeking information on current clinical problems [4]. The orthodontic service provision was influenced by many practitioner characteristics, such as several years since graduation, dentists’ perception of their undergraduate training, and attendance at an orthodontic continuing education course [5].

General dental practitioners are often considered gatekeepers for specialist dental care. They are the ones who typically decide whether, when, and where to refer the patient [6]. It is crucial to make referrals at the right time, as unnecessary appointments can occur if they are made before the patient is ready for treatment. Conversely, if referrals are made after the ideal time, the treatment may become more complicated and time-consuming [7].

The patient waiting list is a significant issue in publicly funded dental clinics. To prevent a longer waiting list, it is essential to make appropriate referrals, which can save time for both patients and clinicians. Priority should be given to those with a greater need for orthodontic treatment, as determined by various orthodontic indices and methods [8].

The IOTN (Index of Orthodontic Treatment Need) is the most commonly used index. It was developed by Brook and Shaw [9]. Then, Richmand et al. [10] introduced the esthetic component (AC) of IOTN. Together, they classify patients into three broad groups: no treatment need; possible treatment need/borderline need; and definite treatment need.

Many research works [11,12,13,14,15,16,17,18,19,20,21,22,23,24] have assessed final-year dental students’ and general dentists’ diagnostic skills and approaches for different malocclusions. The diagnostic skills of undergraduate students regarding different types of malocclusion have been tested by studies [11,13,15].

The present study aims to evaluate the diagnostic skills and treatment approaches of undergraduate dental students by getting them to answer a prepared questionnaire with different malocclusion photos and radiographs, ranging from mild to severe, and assess whether they can diagnose the orthodontic problem considering the severity of the malocclusion and the patient’s age and then refer patients correctly for orthodontic treatment in the dental teaching hospital.

## 2. Materials and Methods

The scientific and ethical committees at the College of Dentistry, Al-Iraqia University, approved the execution of this cross-sectional study during the second session on 7 April 2023.

A comprehensive analysis of clinical competence of final-year dental students was assessed using a paper-based survey which was adopted from that of Yilmaz et al. [18].

At the beginning, the questionnaire was tested and validated primarily by 10 expert orthodontists. This survey was administered to the final-year dental students in the College of Dentistry/Al-Iraqia University as an exam lasting 30 min using the photos and radiographs (lateral cephalometric and orthopantomography) of five previously treated patients.

These cases were selected from two textbooks [25,26] with their full information and ideal treatments, which were planned according to photographic, model, and radiological analyses as the Class I malocclusion, Class II cases (two camouflage treatment cases), and Class III cases (face mask treatment and orthognathic surgery); see Appendices A.1–A.5 (Case 1–5). These cases were selected based on the variety of the causes of the malocclusion and treatment required with full documentation. These cases were assumed to represent a valid diagnostic challenge for the students. From an ethical standpoint, the consent of the book authors was obtained via e-mail to include these pictures in this research.

One hundred and sixty-five final-year dental students were asked to evaluate each case and decide whether the patient needed orthodontic treatment, orthognathic surgery, or neither of these. Moreover, they were asked about the reason for the surgical treatment need, whether irregular teeth or an unesthetic profile. Finally, they should know the reason for the unesthetic profile. The photos and radiographs were presented to the students, and they answered the questionnaire at the same time; then, the answers were compared to the ideal answers presented in the textbooks.

### 2.1. Questionnaire

**1.**  Is orthodontic treatment essential for this patient?
  **a.**  Yes.
  **b.**  No.
**2.**  If your answer is Yes, is orthognathic surgery necessary for this patient?
  **a.**  Yes.
  **b.**  No.
**3.**  If your answer is Yes, what are the causes for this patient needing orthognathic surgery?
  **a.**  Unaesthetic profile and alignment of teeth.
  **b.**  Unaesthetic alignment of teeth.
**4.**  If your answer is (A), what are the factors contributing to the unaesthetic profile?
  **a.**  Protruded mandibular position.
  **b.**  Retruded mandibular position.
  **c.**  Protruded maxillary position.
  **d.**  Retruded maxillary position.

### 2.2. Statistical Analysis

The collected data were analyzed using SPSS software version 25. The frequency and percentage of responses according to the different orthodontic cases and students’ answers regarding types of treatment were tabulated and compared by a Chi-square test. The level of probability was set at 5%.

## 3. Results

Twenty questions regarding five orthodontic cases were answered by one hundred and sixty-five final-stage dental undergraduate students, with a response rate of 100% (Table 1).

Regarding the response to question 1, the distribution of the participant’s answers according to the orthodontic cases and types of treatment showed that the need for orthodontic treatment (correct answer) generally occupied a high percentage for all malocclusion cases: 83%, 73%, 92%, 89%, and 96%, respectively. Class II with camouflage and Class III face mask cases were chosen by 27% and 17% of the students, respectively, to require no treatment, which is incorrect. On the other hand, the remaining cases had somewhat low incorrect answers with a high significant difference, as indicated by the Chi-square test (Table 1).

For question 2, whether any case needs orthognathic surgery or not, 79% of the responses went to orthognathic surgery for class III (correct answer), while low responses indicated that the other cases do not need orthognathic surgery (correct answer). The Chi-square test also showed a highly significant difference. For the cases needing orthognathic surgery, the unesthetic profile and irregular teeth were the major cause (question 3).

Concerning question 4, regarding the factors contributing to an unaesthetic profile, and based on the facial photos and cephalometric analysis attached, the response for the fifth case was 81% for the protruded mandible and 19% for the retruded maxilla, with a high significant difference.

## 4. Discussion

In orthodontics, accurate diagnosis is paramount for proper treatment planning and successful patient treatment outcomes. This includes the identification of the malocclusion, skeletal discrepancies, and an adequate treatment plan formulation for graduating dental students. Studies have shown that dental students lack both confidence and ability when diagnosing in orthodontics, suggesting that there may be gaps in undergraduate education.

The profile of young dental college undergraduates must meet the population’s health needs, so dental colleges are constantly questioning it through the critical evaluation of the quality of university courses [27]. During their clinical career, these dental professionals must be able to identify the occlusal and dentofacial problems in their future patients and refer them for orthodontic treatment [11].

All practitioners are advised to recognize the treatment possibilities and the right timing of application for orthodontic malocclusions [17]. O’Brien et al. [7] found that close to 45% of orthodontic referrals were found to be inappropriate. Some researchers suggest that referral guidelines may assist general dentists in determining the appropriate patients to refer to a specialist [14]. However, O’Brien et al. found no significant change in general dental practitioners’ behavior influenced by referral guidelines. Orthodontic referrals come predominantly from pediatric and general dentists. Orthodontists receive additional education and experience in diagnosing and managing diverse dental and skeletal malocclusions, while education is limited because general dentists are only exposed to orthodontics in dental school [16].

The purpose of this study is to analyze the diagnostic ability of final-year dental students in orthodontic cases and the reported factors affecting students’ ability and to make recommendations for educational enhancement. It is considered the first of its type in Iraq and may be considered a preliminary study for other large-sample-size studies in the future. Additionally, no assessment of the case referral has been conducted yet.

In the present study, the student’s orthodontic skill seems reasonable because the percentage of the selected correct answers of all malocclusion cases was high regarding the need for orthodontic treatment. Moreover, most of the students ensure that they have, to some extent, enough knowledge when they diagnose the cases because a small number of them select incorrect choices regarding no need for orthodontic treatment. This may be attributed to the solid curriculum; additionally, the incorrect answers may be due to the stress of the exam or the limited time. This finding disagreed with that of other studies [28,29].

In an Iranian study about investigating the desire of final-year dental students towards conducting orthodontic treatments in their future profession, Sadeghian et al. [28] found that only 15% of final-year dental students felt confident in their ability to diagnose and treat orthodontic cases upon graduation. A considerable number of the students challenged the number of hours allocated to orthodontics courses and both the theoretical and practical teaching quality. A substantial number of the students wanted to pursue additional orthodontic training following graduation.

On the other hand, Ismail et al. [29] conducted a new qualitative study that indicated that final-year dental students and recent graduates commonly feel ill-equipped in their ability to diagnose orthodontic cases or develop a treatment plan. The participants indicated that their education consisted mainly of theoretical learning rather than practical application, which they felt resulted in low self-confidence when entering a clinical situation. The participants expressed that they would have preferred or found it helpful to have more clinically relevant orthodontic education in their educational experience.

Maxillary growth stimulation, modification, and inhibition must be carried out during the growth period using extra-oral or intra-oral appliances, since a Class III malocclusion is regarded as one of the challenging conditions in diagnosis and treatment [26]. Some researchers thought that undergraduate students should concentrate on the diagnosis of the malocclusion rather than treatment planning [27]; within the current dental curricula, the authors emphasize the importance of changing educational objectives towards training in competence for the management of malocclusions through good recognition and diagnosis to make general dental clinical practice more relevant, as training dental students for treating complex malocclusions is not practical or feasible.

However, this study focuses on both the clinical competence and diagnostic proficiency of dental students, reflecting the interest in training students in dental teaching hospitals. The undergraduate students in the study by Canavero et al. [13] encountered difficulties in diagnosing class II and even found it hard to come up with a clear idea about a basic treatment protocol to correct this malocclusion. While increased overjet was commonly recognized, other features like increased overbite were less frequently identified. This suggests a need for more comprehensive training in recognizing various malocclusion characteristics.

Yalmiz et al. [18] reported that the class II camouflage case that needed treatment was less than other malocclusion cases in the present study. At the same time, class II orthognathic surgery has a higher treatment need, which conflicts with another study [18], with a low percentage of patients indicated for orthognathic surgery.

Students should have a lot of awareness and information about orthognathic surgery, which is considered an elective intervention and most of the time is carried out only for aesthetic reasons, so an accurate interpretation of the photographs and cephalometric X-ray in addition to knowledge about the envelope of discrepancy will aid in diagnosing such cases. In addition to that, the student’s answer regarding the class II camouflage choice that there is no need for orthognathic surgery was very high in this study based on the patient age and cephalometric analysis given, in addition to the right treatment plan, which was the extraction of four first premolars, but orthognathic surgery was indicated for the severe skeletal discrepancy.

Using the Diagnostic Thinking Inventory, an evaluation of clinical reasoning skills in dental students in Karachi showed poor overall performance. The study showed that the students had poor scores in both the knowledge structure and ease of flexibility types of thinking, both directly correlated with diagnostic ability. Notably, the female students were found to have better diagnostic thinking abilities than the male students [30].

Tuncer et al. [20] concluded that case-based orthodontic learning was positively received by final-year dental students, and the greatest difference was observed in motivation, to give feedback regarding orthodontic case diagnosis and treatment planning, without feeling any extra workload, so it is recommended to outline specific steps for improving the diagnostic training of dental students, such as integrating more practical experience, case-based learning, or access to digital and 3D diagnostic tools. Group discussion and frequent exams are necessary to test the competence of future dentists in diagnosing and referring orthodontic cases.

There are many factors that influence the diagnostic proficiency among different final-year dental students; differences in the orthodontic curriculum across dental schools have contributed to discrepancies in proficiency for students. In a scoping review conducted by Raghavan et al. [31] regarding differences in orthodontic curricula, the authors found substantial differences in not only learning outcomes but also content and assessments. The authors recommended standardization in the curriculum to be congruent with professional standards and to ensure student readiness for the clinic.

In addition, a difference in emphasis on theory and practical experience has been a challenge in the development of diagnostic skills. Students noted that practical sessions emphasized tasks such as wire bending and there was little time spent on real world diagnostic tasks, which restricted the development of clinical reasoning and decision making [29].

The other factor is clinical exposure and hands-on experience. Clinical exposure is fundamental in developing diagnostic confidence. At Cardiff University, a study found that 69% of fourth-year dental students felt confident managing orthodontic emergencies after supervised patient treatment. Students indicated the need to combine their theoretical knowledge with practical clinical experience and indicated the value of experiencing a supported clinical learning environment [32].

However, many students have reported inadequate experience in clinical exposure during their undergraduate dental education. Restricted opportunities to engage in the full-cycle treatments related to patient diagnosis and treatment planning obstruct the learning of critical capacity development. Improving clinical rotations and experiences with a case mix can improve a student’s diagnostic capability [31].

The last factor is the assessment and feedback mechanisms. In order to develop diagnostic skills, assessment and feedback are essential. Research on assessment practice suggests traditional assessment may not adequately measure students’ diagnostic skills. Formative assessments such as case-based discussions or reflective/situational exercises provide students with feedback and points for improvement.

When students have some baseline knowledge, there is the opportunity to add some simulation and roleplay practicum work to enhance clinical reasoning skills. Simulation offers students a safe place to practice how to diagnose more complex cases and share diagnostic processes that encourage confidence and competence.

One of the limitations of this study is conducting it at one college with a relatively small sample size, which might produce a latent bias. Moreover, the excitement of students’ graduation may have impacted their outcomes, so it is better to conduct similar studies with an increase in the sample size and cases and to include dental colleges across the country to achieve a comprehensive view of the scientific and professional level of future dentists.

There are some recommendations to improve diagnostic proficiency:

**1.**  Curriculum improvement: Dental schools should modify their curricula to maintain an equilibrium of knowledge while offering sufficient opportunity for application. Offering integrated teaching strategies such as lectures with clinical experiences will help students with their practical applications.
**2.**  Standardization of learning goals: Developing standard learning outcomes and competencies in orthodontic education across institutions may aid in further consistency. Additionally, the curriculum can be aligned to regulatory organizations so students will be prepared for professional practice expectations.
**3.**  More clinical exposure: Clinical oversight during diverse clinical experiences with varying malocclusions and treatment planning opportunities will develop students’ diagnostic values. Clinical rotations and mentorships can add to experiences during school and offer clinic and patient care instruction.
**4.**  Innovative teaching styles: The use of simulation-based training, case-based learning, and role playing becomes more essential as students increase clinical reasoning and decision making. Utilizing strategies that encourage realistically replicating their learning and undertaking obstacles to their learning opportunity provides students with opportunities to implement previously learnt concepts and theories in new practical environments.
**5.**  Ongoing assessment: Formative assessment and feedback can put students in a position to develop good self-awareness of strong and weak abilities. It can also contribute to self-directed learning and skill building through ongoing reflective practice.
**6.**  Faculty development: By investing in faculty–student training to embrace innovative teaching and assessment strategies, the faculty will improve their changing role in orthodontic education for students. The faculty should be both professionally and pedagogically equipped to create and use active learning opportunities while providing mentoring.

Finally, the findings suggest that final-year dental students can generally identify when orthodontic treatment is appropriate and see gross skeletal anomalies, but they struggle with diagnostic detail, such as differentiating the components of the jaw and the difference between normal variations in the population versus borderline cases. The educational implications include the following:

**1.**  More structured clinical exposure to borderline cases.
**2.**  More training in cephalometrics and reading facial profiles.
**3.**  More use of 3D imaging and visual devices to reinforce diagnosis.

The data overall highlight a useful diagnostic checkpoint and identify the points of strength and gaps in education for final-year dental school students’ orthodontic training.

## 5. Conclusions

The clinical competence and diagnostic skills of the undergraduate dental students for all different malocclusion cases that ranged from mild to severe appear good; they have the ability to diagnose orthodontic cases considering the severity of the malocclusion and the patient’s age. Class II and III malocclusion cases are highly selected to need orthognathic surgery. The unesthetic profile and irregular teeth were the primary reasons for referring all malocclusion cases for orthognathic surgery treatment.

## Figures and Tables

**Table 1:** Frequency distribution and comparison of the responses of the participants.

Questions	Answers	Case No. 1 Class III Face Mask Treatment	Case No. 2 Class II Camouflage Treatment	Case No. 3 Class I	Case No. 4 Class II Camouflage Treatment	Case No. 5 Class III Orthognathic Surgery
**Q1**	**Need orthodontic treatment**	137 (83%)	120 (73%)	151 (92%)	147 (89%)	159 (96%)
	**No need** **for orthodontic treatment**	28 (17%)	45 (27%)	14 (8%)	18 (11%)	6 (4%)
	***p*-value**	≤0.001	≤0.001	≤0.001	≤0.001	≤0.001
**Q2**	**Need** **orthognathic surgery**	9 (7%)	13 (11%)	22 (15%)	23 (16%)	126 (79%)
	**No need** **for orthognathic surgery**	128 (93%)	107 (89%)	129 (85%)	124 (84%)	33 (21%)
	***p*-value**	≤0.001	≤0.001	≤0.001	≤0.001	≤0.001
**Q3**	**Unesthetic profile** **and irregular teeth**	9 (100%)	11 (85%)	17 (77%)	23 (100%)	124 (98%)
	**Irregular ** **teeth**	0 (0%)	2 (15%)	5 (23%)	0 (0%)	2 (2%)
	***p*-value**	-	0.022	0.017	-	≤0.001
**Q4**	**Protruded** **mandible**	0 (0%)	0 (0%)	0 (0%)	1 (4%)	101 (81%)
	**Retruded** **mandible**	8 (89%)	3 (27%)	5 (29%)	6 (26%)	0 (0%)
	**Protruded** **Maxilla**	0 (0%)	8 (73%)	12 (71%)	16 (70%)	0 (0%)
	**Retruded** **Maxilla**	1 (11%)	0 (0%)	0 (0%)	0 (0%)	23 (19%)
	***p*-value**	0.039	0.277	0.143	≤0.001	≤0.001

**Figure A1:**
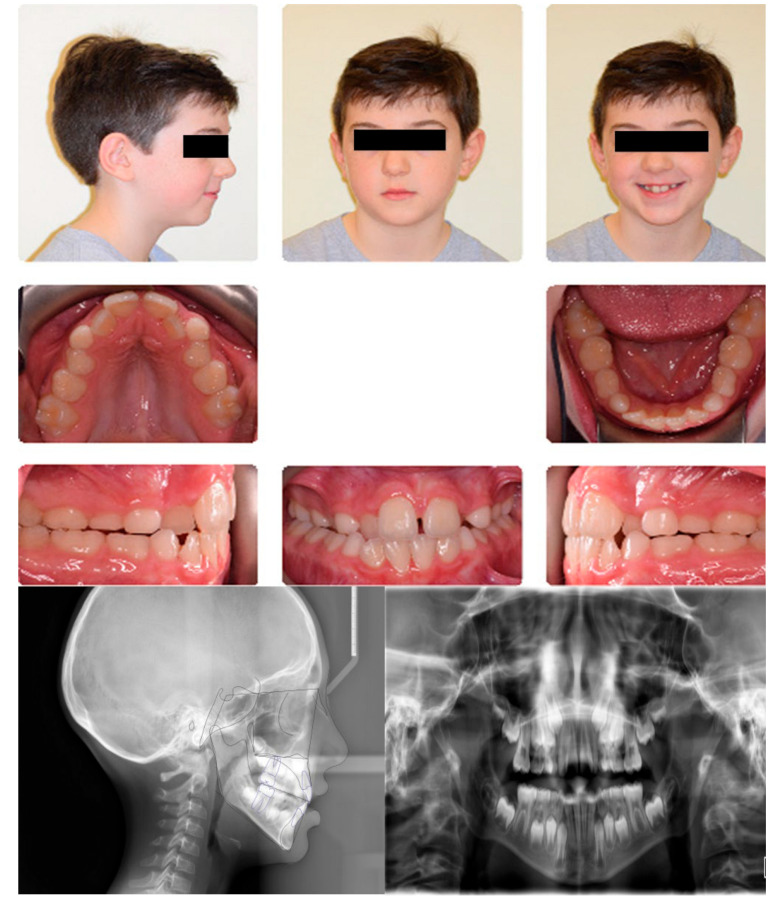
Full records of the patient.

**Figure A2:**
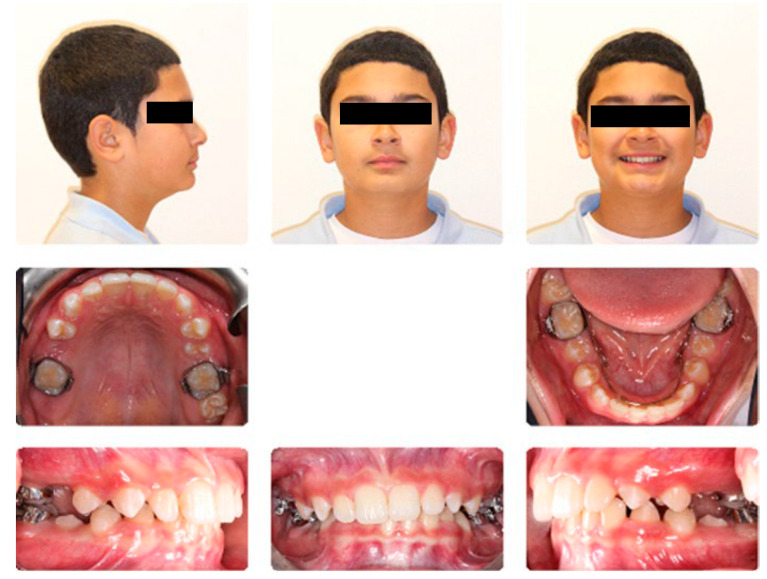

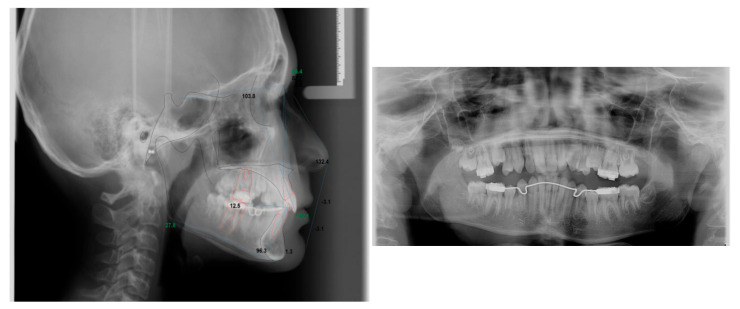
Full records of the patient.

**Figure A3:**
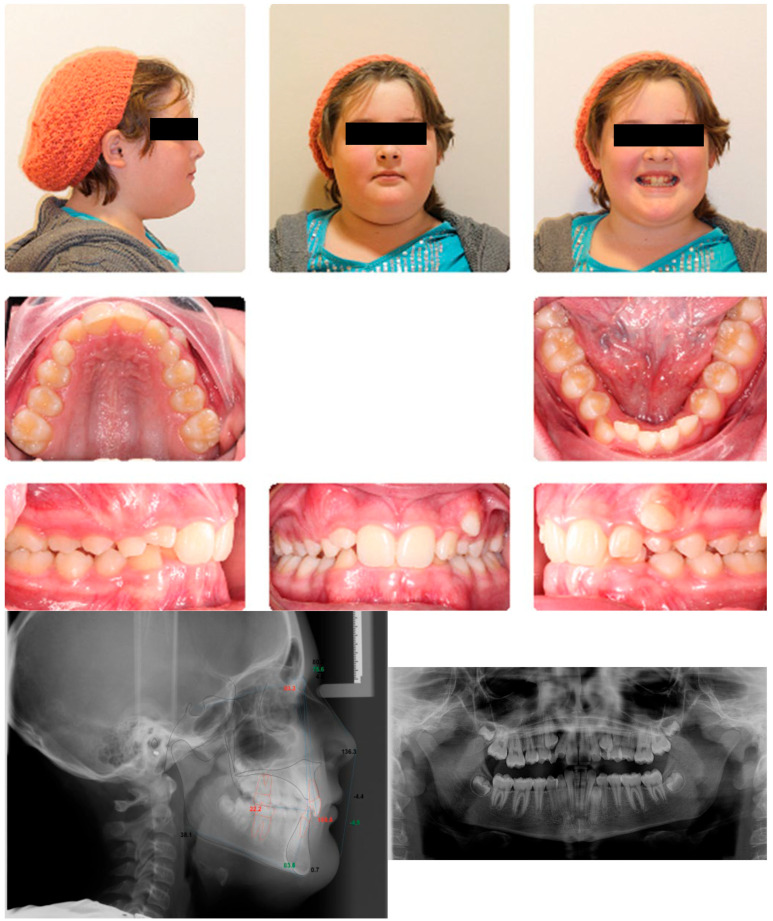
Full records of the patient.

**Figure A4:**
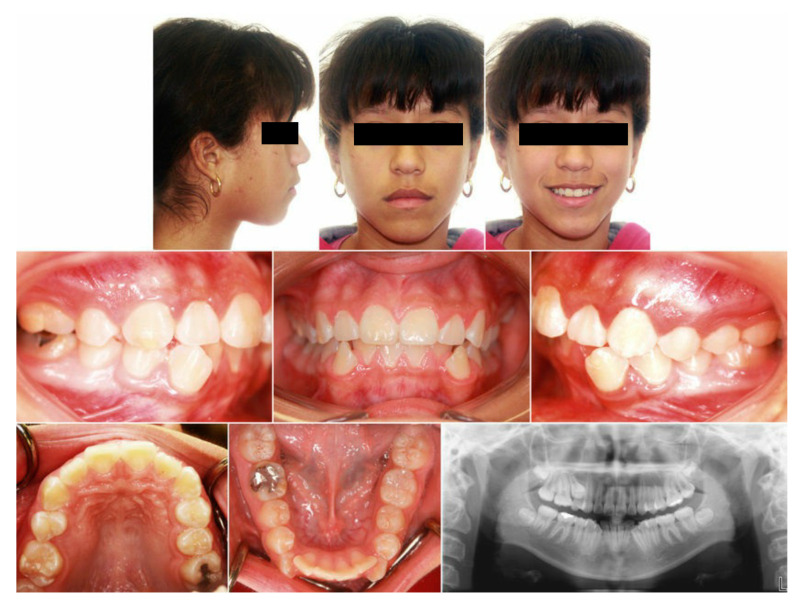
Full records of the patient.

**Figure A5:**
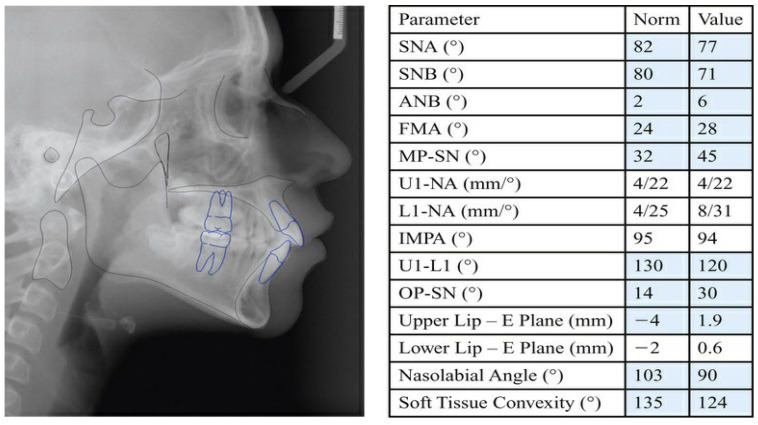
Cephalometric analysis of the case.

**Figure A6:**
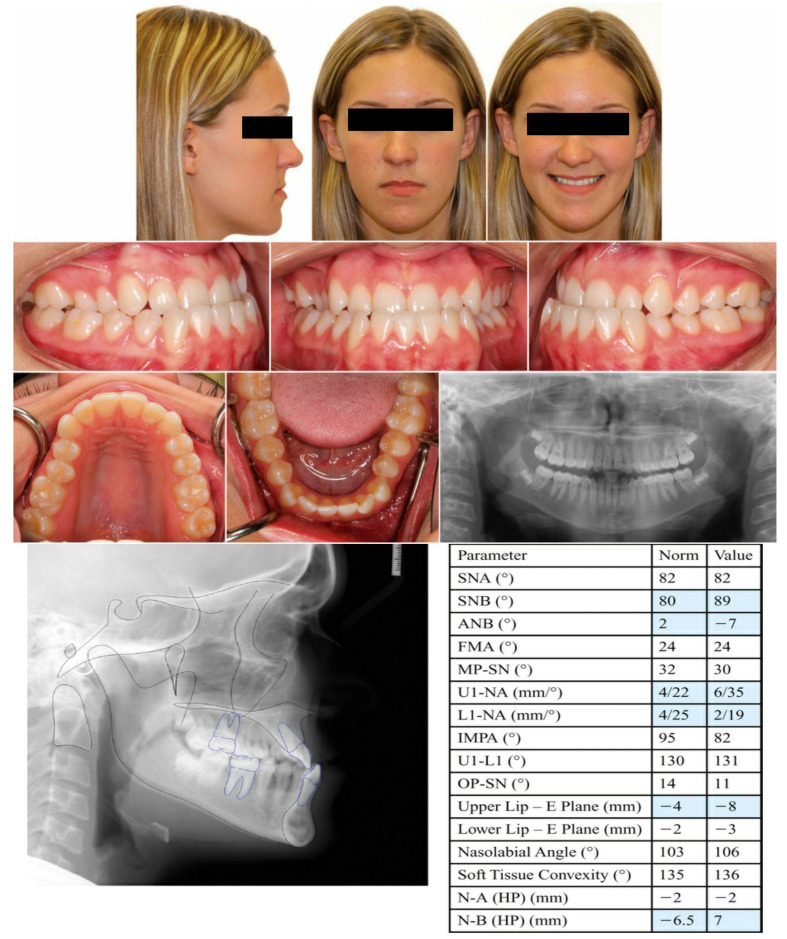
Full records of the patient and cephalometric analysis of the case.

## Data Availability

The data are available on request from the corresponding author.

## Appendix A. (Clinical Cases Exam)

### Appendix A.1. Case 1

**Table A1:** Demographic data and cephalometric analysis of the case.

Age	8 years	Over Jet = 1 mm
Sex	Male	Over bite = 1 mm
Breathing	Nasal	
Cephalometric analysis		
	Normal values	Patient values
SNA	80	78.6°
SNB	78	75.9°
ANB	2	+2.7°
FMA	21	30.2°
SN-GoGn	32	41.4°
Maxillary incisor to SN	105	97.4°
Mandibular incisor to GoGn	95	85.5°
Soft tissue		
Lower lip to E-plane	–2.0 mm	+0.4 mm
Upper lip to E-plane	–1.6 mm	–3.5 mm

### Appendix A.2. Case 2

**Table A2:** Demographic data and cephalometric analysis of the case.

Age	11 years	Over Jet = 3 mm
Sex	Male	Over bite = 5 mm
Breathing	Nasal	
Cephalometric analysis		
	Normal values	Patient values
SNA	80	86.4°
SNB	78	82.7°
ANB	2	+3.7°
FMA	21	23°
SN-GoGn	32	27.8°
Maxillary incisor to SN	105	103.8°
Mandibular incisor to GoGn	95	96.3°
Soft tissue		
Lower lip to E-plane	–2.0 mm	−3.1 mm
Upper lip to E-plane	–1.6 mm	−3.1 mm

### Appendix A.3. Case 3

**Table A3:** Demographic data and cephalometric analysis of the case.

Age	11 years	Over Jet = 4 mm
Sex	Male	Over bite = 8 mm
Breathing	Nasal	
Cephalometric analysis		
	Normal values	Patient values
SNA	80	80.2°
SNB	78	75.6°
ANB	2	+4.3°
FMA	21	24.2°
SN-GoGn	32	38.1°
Maxillary incisor to SN	105	83.3°
Mandibular incisor to GoGn	95	83.3°
Soft tissue		
Lower lip to E-plane	–2.0 mm	−4.9 mm
Upper lip to E-plane	–1.6 mm	−4.4 mm

### Appendix A.4. Case 4

**Table A4:** Demographic data.

Age	14 years	Over Jet = 3 mm
Sex	Female	Open bite = 4 mm
Breathing	Nasal	

### Appendix A.5. Case 5

**Table A5:** Demographic data.

Age	17 years	Over Jet = −3 mm
Sex	Female	Over bite = 3 mm
Breathing	Nasal	
